# Is Variety the Spice of Life? An Experimental Investigation into the Effects of Species Richness on Self-Reported Mental Well-Being

**DOI:** 10.1371/journal.pone.0170225

**Published:** 2017-01-20

**Authors:** Lukas J. Wolf, Sophus zu Ermgassen, Andrew Balmford, Mathew White, Netta Weinstein

**Affiliations:** 1 School of Psychology, Cardiff University, Cardiff, United Kingdom; 2 School of Geosciences, University of Edinburgh, Edinburgh, United Kingdom; 3 Department of Zoology, University of Cambridge, Cambridge, United Kingdom; 4 Psychology Applied to Health & the Environment, University of Exeter Medical School, Exeter, United Kingdom; Cornell University College of Veterinary Medicine, UNITED STATES

## Abstract

Losses in biodiversity and trends toward urbanisation have reduced people’s contact with biodiverse nature, yet the consequences for mental well-being are not well understood. Here, we demonstrate that greater plant and animal species richness in isolation causes an improvement in mental well-being. To do so, the present research experimentally manipulated species richness and assessed widely-used indicators of mental well-being. Participants viewed short videos of either high or low tree (Study 1) or bird (Study 2) species richness and reported on positive (i.e., vitality, positive affect) and negative (i.e., anxiety) indicators of mental well-being. Building on Study 1, Study 2 included an urban environment as a reference treatment and explored the role of giving participants information on the presented environment. We find that, in line with expectations, watching videos containing greater species richness consistently leads to higher mental well-being. We discuss findings in light of the importance of connecting people to biodiverse environments.

## Introduction

According to a recent United Nations census, approximately 75% of the population in developed countries lives in urban areas [1]. Even world-wide there are now more people living in urban than rural areas, a trend which is expected to continue over the next decades [1]. At the same time, species extinction is occurring at 100 to 1000 times above background rates seen in the fossil record [2–4] with no indication of it slowing down [5]. These trends suggest that individuals are at risk of becoming increasingly detached from rich natural environments [6,7], a worrisome development given that extensive research points to the beneficial mental and physical well-being effects of exposure to nature and the detrimental effects of urban built surroundings [7,8]. Even though urban areas attempt to compensate for this by providing greenspaces such as parks, urban areas are generally associated with reduced levels of biodiversity (defined here as the richness or diversity of plant and animal species) [9].

This loss of contact with biodiverse spaces is important because there is tentative evidence suggesting that natural environments high in biodiversity or species richness provide additional benefits for people’s mental well-being compared to natural environments low in biodiversity [10–12]. Despite these promising findings, previous work has largely been correlational and unable to draw causal conclusions about the effects of biodiversity [13]; it is hence unclear whether contact with biodiverse environments directly improves mental well-being. The purpose of the present research is to explore the causality of the association between biodiversity and mental well-being through a controlled experimental setting, and hence to improve our conceptual understanding of a potentially important ecosystem service. To measure mental well-being, defined here as the positivity of one’s affective states [14], we used short versions of well-validated measures that operationalise this construct in terms of positive affect, vitality, and lower anxiety. We have selected these indicators of mental well-being because they have been used previously as important outcomes of contact with nature and have been associated with other short-term and long-term, mental and physical well-being outcomes [15–20].

### Benefits of natural environments for mental well-being

According to the biophilia hypothesis [21], people have an innate affinity toward plants and animals. It has been suggested that this affinity is an adaptive response to living in the sorts of natural environments in which humans evolved, and that certain plants and animals still trigger automatic approach (or avoidance) motivations and accompanying emotions [21,22] (but see [23]). In support of this hypothesis, evidence has consistently shown the beneficial effects of natural environments on people’s mental well-being [22,24,25]. Indeed, a recent meta-analysis across 32 studies showed that exposure to natural environments yielded a moderate increase in positive emotions and a smaller but reliable decrease in negative emotions [8]. Important for the present research, this meta-analysis also indicated that whereas exposure to real nature yielded stronger effects than exposure to laboratory simulations (e.g., videos), the effects of simulated natural settings were still robust. In sum, there is strong evidence that natural environments benefit mental well-being more than urban built ones.

### Benefits of biodiversity for mental well-being

The biophilia hypothesis discussed above could suggest that one characteristic of natural environments, their biodiversity, benefits people’s mental well-being. That is, from an evolutionary perspective [21], it could be expected that exposure to more biodiverse environments improves mental well-being because humans could be assumed to prefer the biologically rich environments in which we evolved.

There is empirical support for the prediction that humans prefer more rich, biodiverse environments. For instance, Lindemann-Matthies et al. [26] found that individuals consistently preferred arrays of pots with higher plant diversity, although the findings differed as to whether perceived diversity or actual diversity had a stronger impact. However, while this study and others [27,28] have shown that biodiverse environments are appealing to people, they did not test whether these environments improve people’s mental well-being.

Addressing this question, Lovell et al. [13] stated in a recent systematic review that the evidence on the benefits of biodiversity on mental and physical well-being is so far inconclusive. This systematic review included ten quantitative studies that considered biodiversity, species richness, or areas protected for their biodiversity in relation to mental well-being. While several studies found positive results [10,11,29–32], three revealed no relationship [33–35] and Dallimer et al. [12] obtained mixed results.

In addition to showing mixed evidence, the literature on the link between biodiversity and mental well-being reveals three further issues that the present research seeks to address. First, in the most impactful studies in this literature, Fuller et al. [10] and Dallimer et al. [12] used constructs that assess specific perceptual experiences associated with the particular greenspace studied (e.g., place attachment) or that are relatively indirect indicators of well-being (e.g., ‘reflection’ or the ability to gain perspective) [36] rather than directly testing general indicators of mental well-being. The present research addresses this issue by using short versions of validated measures to assess positive affect, vitality or energy, and anxiety—constructs that have been employed previously to assess mental well-being and that have both short-term and long-term implications for well-being and functioning [18–20].

Second, studies testing species richness in isolation have so far been correlational, making it difficult to draw causal conclusions about the effects of biodiversity. For example, it may be that those who report higher mental well-being are more likely to visit areas high in biodiversity. While some experimental studies have compared the benefits of different natural environments on people’s mental well-being [34,37–40], like many of the field studies [10,12], they were also unable to unpack the relative importance of species richness vs. species abundance (i.e., the absolute numbers of animals and plants present). Moreover, many of the field studies collected data on species richness several months before they collected data on mental well-being and there is good reason to speculate (e.g., seasonal variation) that the particular species richness present during times of human exposure was not the same as when it was originally measured. These issues were highlighted in Lovell et al.'s [13] systematic review in which the authors concluded that there is a need for robust experimental and controlled designs; in line with this need, the present research addresses this shortcoming in experimental and controlled studies to test the causality of associations between biodiversity and mental well-being.

A final issue is that findings are so far inconsistent as to whether perceived or actual biodiversity yields beneficial outcomes [10,12,26]. Presumably, people generally have limited ability to identify species [12,26], a limitation which may be reduced through educating them about natural spaces. The importance of education has been highlighted by Dallimer et al. [12], who found that participants with better knowledge of wildlife gave more accurate estimates of actual biodiversity, and by Shwartz et al. [28] who found that garden visitors noticed more flower diversity when their awareness of biodiversity was increased through information and visitor-focused activity days. To examine whether education enhances the beneficial effects of biodiversity on people causally, the present research explores the role of giving participants relevant information about built and natural environments.

### Objectives and present research

Informed by the literatures discussed above, the present research aims to examine a causal model testing biodiversity and wellness, using direct measures of mental well-being, and to explore the effects of giving participants information about biodiverse spaces. To do so, we manipulated species richness in videos in two controlled experiments. We measured participants’ mental well-being by assessing positive affect, vitality, and reported anxiety, all indicators which have been robustly linked to long-term outcomes [18–20]. In a second study, we also manipulated education about built and natural environments.

## Study 1 –Method

### Participants

We conducted a power analysis to determine the required sample size. Although previous studies testing the link between biodiversity and well-being found a large effect size [10,12], it was difficult to prefigure what the effect sizes would be given the different design and outcome measures used in the present study. Basing our power analysis on a medium effect size (Cohen’s *d* = 0.5), the required sample size was 126 participants to achieve a power of .80.

We recruited through Mechanical Turk, an online crowdsourcing platform that has been frequently used as a recruitment tool for research. Previous studies comparing data from Mechanical Turk to data from traditional recruitment methods have shown that Mechanical Turk samples are more demographically diverse and that the results are generally consistent and robust [41–43]. We recruited 140 participants living in the US (81 men; 18–61+ years of age; *M*_age_ = 36 years) to take part in an online study and we randomly assigned them to a low (*n* = 56) or high (*n* = 84) biodiversity treatment. Unequal sample sizes resulted from random assignment procedures, but were not of particular concern given we planned to analyse data with analyses of variance, which are less vulnerable to such differences in samples [44]. Participants completed the study in less than 10 minutes and received $1 in exchange for their participation. All participants completed the study.

### Procedure

Participants completed surveys in their own environment and on their own device. To ensure higher data quality, we instructed participants at the beginning of the survey to find a quiet and private space without distractions, and provided a standard set of instructions to prepare for video and audio. Those in the low biodiversity treatment viewed a short (101–112s; *Mean =* 106s) video of one of four species of trees (randomly assigned): oak (*Quercus*), redwood (*Sequoia*), spruce (*Picea*), willow (*Salix*); videos were kept short to reduce participant burden and ensure all participants watched the video in its entirety. Different species were used to ensure that the particular characteristics of any one species did not drive effects in this treatment. Participants in the high biodiversity treatment viewed a video of comparable length, showing all four tree species. To ensure the manipulation was of the number of species only, we showed participants in both treatments a comparable number of individual trees, all seen from varied angles and with different backgrounds.

In both treatments, participants read instructions to help immerse them in this artificial environment, which were adapted from Weinstein et al. [45]: “As you view this video, imagine you are taking a walk outside. Look around, noticing aspects of the environments. Let yourself take in the things you see, the way you might if you were actually taking a stroll outside.” Directly after viewing the video, participants indicated their mental well-being.

### Measures

We measured positive affect, vitality, and anxiety as indicators of mental well-being, because these are important outcomes of contact with nature associated with short-term and long-term well-being outcomes [15–20]. We used shortened versions of the scales to reduce participant burden. Participants were presented with 28 mental well-being items in total. Each scale was presented on a separate screen, with the order of items and the order of scales randomised.

To measure positive affect, we administered the Positive and Negative Affect Schedule (PANAS) [18]. We used 12 items of the original 20 items to reduce participant burden. The PANAS is a validated measure that has been linked to higher mental and physical well-being [18,46] and it has shown benefits of walking in nature or being presented with natural settings [16]. Participants indicated for 12 adjectives (e.g., enthusiastic, upset) to what extent they felt this way at that moment on a scale from 1 (not at all) to 5 (extremely). After reverse-scoring negative items, the PANAS showed acceptable reliability (α = .82).

To measure vitality, we administered the six-item version of the Subjective Vitality Scale (SVS) [19], based on previous validation work showing better internal consistency in this version [47]. The SVS has been associated with mental and physical well-being indicators [19] and it has revealed positive effects of imagining or actually being in natural outdoor settings [15]. Participants responded to items (e.g., “at this moment I feel alive and vital”, “I have energy and spirit”) using a scale from 1 (not at all) to 5 (very true). This measure was internally consistent (α = .92).

We measured participants’ reported anxiety with the State Trait Anxiety Inventory (STAI) [20]. To reduce participant burden, we used 10 of the original 20 items, similar to other 10-item versions used in previous studies [48,49]. The STAI has been associated with various mental and physical well-being indicators [50] and it has for instance revealed reduced anxiety when having an office view of nature compared to an office view of the city [17]. The STAI comprises items such as “I feel tense” and “I feel relaxed” (reversed), paired with a scale from 1 (not at all true) to 5 (very true). The STAI was internally consistent (α = .88).

### Ethical issues

The research has been conducted according to the principles expressed in the Declaration of Helsinki. This study has been approved by the Faculty of Science and Health Ethics Committee at University of Exeter. Participants gave written informed consent to take part in the study, they were given the right to withdraw at any moment, and they were assured of the anonymity of the data.

## Study 1 –Results

### Correlations among mental well-being constructs

All outcomes were highly correlated. Vitality was associated with both higher positive affect (*N* = 140, *r* = .74, *p* < .001) and lower anxiety (*r* = -.52, *p* < .001), and higher positive affect was associated with lower anxiety (*r* = -.50, *p* < .001).

### Effects on mental well-being

We conducted a MANOVA with biodiversity (high vs. low) as the between-subject factor on the dependent variables positive affect, vitality, and anxiety. The multivariate test was significant (*F*[3,136] = 3.59, *p* = .015, *η*^*2*^ = .07), allowing us to test the three mental well-being measures in separate between-subjects t-tests.

**Fig 1:**
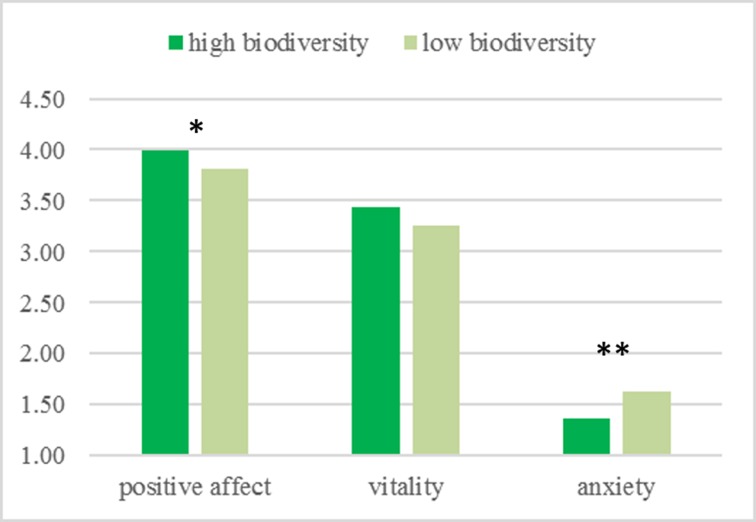
Mean ratings for the factor environment on positive affect, vitality, and anxiety in Study 1. Possible ratings on all measures range from 1 to 5. Higher mean scores indicate higher positive affect, vitality, and anxiety. * significant at .05 level ** significant at .01 level.

The effect of biodiversity was significant on positive affect (*t*[138] = 2.00, *p =* .048) and on anxiety (*t*[138] = -3.03, *p =* .003). As can be seen in Fig 1, participants who saw the high biodiversity video reported higher positive affect (*M =* 3.99, *SE =* 0.06) and lower anxiety (*M =* 1.36, *SE =* 0.05) than participants who saw the low biodiversity video (*M =* 3.81, *SE =* 0.06; *M =* 1.63, *SE =* 0.06). Vitality was not linked to biodiversity in this study (*t*[138] = 1.12, *p =* .27; high biodiversity: *M =* 3.43, *SE =* 0.12; low biodiversity: *M =* 3.25, *SE =* 0.10).

## Study 1 –Summary

Study 1 manipulated biodiversity by comparing natural settings with either a single tree type (low species richness) or four tree types (high species richness). We found that participants who viewed videos showing higher species richness reported more positive affect and less anxiety than those who viewed videos showing low species richness.

Although this finding provided initial support that exposure to more species-rich environments promotes mental well-being, Study 1 had limitations that we aimed to address in a second study. First, Study 1 does not anchor well into the much larger body of work comparing natural environments of any type to urban spaces [22,24,25]. In Study 2, we therefore included an urban built setting as a reference treatment, allowing us to assess the benefits of biodiversity relative to the better established effects of urban vs. natural settings. Second, we measured baseline mental well-being to more accurately capture the variance that directly resulted from exposure to the manipulation, allowing for a more sensitive test of the effects. Third, we aimed to improve generalizability of our findings by employing a different set of natural stimuli–in this case low or high numbers of bird species. Finally, given previous work has shown that educating people might help them to better appreciate natural spaces [12,28], we tested the added effects of educating participants about low biodiverse, high biodiverse, and urban environments.

## Study 2 –Method

### Participants

As in Study 1, we conducted a power analysis to determine the required sample size. We based our power analysis on the effect on positive affect in Study 1 (Cohen’s *d* = 0.35) given that it was the smallest significant finding. The required sample size was 259 participants to achieve a power of .8.

We recruited 264 Americans (137 women; 18–61+ years of age; *M*_age_ = 35 years) to complete the study online on Mechanical Turk. Participants were randomly assigned to one of six treatments, using a 3 (environment: urban built, low biodiversity, high biodiversity) X 2 (information: yes, no) design. One-hundred and thirty-four participants were assigned to the no information treatment (*n*_urban built_ = 42; *n*_low biodiversity_ = 41; *n*_high biodiversity_ = 51) and 130 participants were assigned to the information treatment (*n*_urban built_ = 56; *n*_low biodiversity_ = 40; *n*_high biodiversity_ = 34). Participants completed the study in less than 10 minutes and received $1 in exchange for their participation. All participants completed the study.

### Procedure

Participants who were assigned to the information treatment read four simple text passages related to their environment treatment. Following this, all participants completed baseline measures of positive affect, vitality, and anxiety. Participants were then assigned to watch a video of their environment treatment, which was paired with the immersion instructions used in Study 1. As in Study 1, participants then completed follow-up measures of positive affect, vitality, and anxiety.

### Materials

#### Information treatment

Five text passages (each 26–34 words) were presented on separate pages. In the two nature treatments, participants read five facts about the birds they would later see, e.g., “Did you know? If the Chaffinch is not exposed to the adult male’s song during a certain critical period after hatching, it will never properly learn the song”. While participants in the low biodiversity treatment saw five facts about one bird species they would later see, participants in the high biodiversity treatment received five facts about each of the five species. Those assigned to the urban videos learned five facts which were of comparable length and formatting, which related to the objects in these videos (e.g., did you know? Bus is a clipped form of the Latin word *omnibus*. It appeared in Paris in 1819–1820 as “*voiture omnibus”* meaning “carriage for all”). Each fact was paired with an image of the bird species or the urban object.

#### Choice of bird species

We presented five passerine species—the European Robin (*Erithacus rubecula*), Dunnock (*Prunella modularis*), House Sparrow (*Passer domesticus*), Common Chaffinch (*Fringilla coelebs*) and Song Thrush (*Turdus philomelos*). Species were chosen on the basis of displaying modest colouration and similar body shape, such that they would all be comparably memorable to participants.

#### Videos

Participants in the low biodiversity treatment videos were presented with one of five videos each showing different individuals from a single species. In the high biodiversity treatment, participants were presented with one of three videos (each presenting species in a different order to avoid order effects), showing all five chosen species using footage taken from the low biodiversity treatment. In the urban treatment, participants saw one of three different videos, each showing identical footage presented in a different order. The videos depicted objects commonly found in an urban landscape (e.g., street sign, bus, traffic cone), which were selected to focus participants’ attention to their environment in the same way that one would focus on a bird in a natural space (i.e., taking an interest in one object rather than the landscape). Comparable numbers of individuals and objects, angles, and light levels were used in all treaments.

The bird video clips were acquired from RSPB's Big Garden Birdwatch [51] and the Internet Bird Collection database [52]. We selected 10 clips for each species that fulfilled the following criteria: the clip contained a single, high-resolution individual of the chosen species performing a low-intensity activity, with a natural background. Backgrounds contained two or fewer obviously identifiable plant species and no other animals in order to control for non-focal biodiversity. We ensured that the dominant sound in each clip was the call of the focal species, where necessary overlaying a soundtrack of the focal species’ call using recordings acquired from xeno-canto [53]. Urban videos were selected for comparable qualities and included sounds that were typical for the object being shown (e.g., bus) or included a very soft background presentation of typical urban sounds. The length of all of the final videos ranged from 116 seconds to 124 seconds (*M =* 119s).

#### Mental well-being

We assessed the same mental well-being constructs as in Study 1 at baseline and at follow-up. We reduced the number of items by selecting eight items of the PANAS scale to assess positive affect (α = .74), three items of the SVS to assess vitality (α = .93), and six items of the STAI to assess reported anxiety (α = .90). We have opted to use shorter baseline measures given that previous research has already successfully employed similar shortened versions of these validated scales [15,54,55], and to reduce participant burden. In the more extensive follow-up measures, we used the same items for the PANAS scale (α = .83), the SVS (α = .93), and the STAI (α = .91) as described in Study 1. Hence, at baseline, participants were presented with 17 items in total and at follow-up, participants were presented with 28 items in total. Both at baseline and at follow-up, each scale was presented on a separate screen, with the order of items and the order of scales randomised. The baseline and follow-up measures correlated very highly for the PANAS (*r* = .87), SVS (*r* = .84), and STAI (*r* = .81) scales, indicating they indeed measured highly similar constructs at both measurement moments.

### Ethical issues

This study has been approved by the Faculty of Science and Health Ethics Committee at University of Exeter. We followed the ethical guidelines as described in Study 1.

## Study 2 –Results

### Correlations among mental well-being constructs

For the baseline measures, positive affect was associated with higher vitality (*N* = 264, *r* = .71, *p* < .001) and lower anxiety (*r* = -.69, *p* < .001) and vitality and anxiety were negatively correlated (*r* = -.58, *p* < .001). For the follow-up measures, positive affect linked to more vitality, *r* = .75, *p* < .001, and lower anxiety (*r* = -.66, *p* < .001) and vitality and anxiety were also negatively associated (*r* = -.50, *p* < .001).

### Effects on mental well-being

#### Multivariate test

We conducted a 3 (environment: urban built vs. low biodiversity vs. high biodiversity) x 2 (information: yes vs. no) MANCOVA on the post-measures of positive affect, vitality, and reported anxiety, with the pre-measures of positive affect, vitality, and anxiety as covariates. Multivariate tests for environment (*F*[6,506] = 6.40, *p* < .001, *η*^*2*^ = .07), information (*F*[3,253] = 3.75, *p* = .012, *η*^*2*^ = .04), and their interaction (*F*[6,506] = 2.78, *p* = .012, *η*^*2*^ = .03) were significant, allowing us to test the three mental well-being measures in separate ANCOVAs (Table 1).

**Table 1:** Descriptive statistics for the factors environment and information on positive affect, vitality, and anxiety.

			Positive affect		Vitality		Anxiety	
			Pre	Post	Pre	Post	Pre	Post
Environ.	Information	*n*	*M* (*SD*)	*M* (*SD*)	*M* (*SD*)	*M* (*SD*)	*M* (*SD*)	*M* (*SD*)
Urban	No	42	3.76	3.6	3.16	2.85	2.27	2.09
			(0.63)	(0.62)	(1.06)	(0.97)	(0.98)	(0.82)
	Yes	56	4.03	3.8	3.65	3.1	1.87	1.84
			(0.44)	(0.46)	(0.85)	(0.76)	(0.62)	(0.67)
	Total	98	3.92	3.72	3.44	3	2.04	1.95
			(0.55)	(0.54)	(0.97)	(0.86)	(0.81)	(0.74)
Low	No	40	3.67	3.6	3.12	2.76	2.27	2.14
			(0.66)	(0.66)	(1.22)	(1.00)	(1.01)	(0.88)
	Yes	41	3.83	3.7	3.01	2.99	2.34	1.89
			(0.67)	(0.64)	(1.22)	(1.08)	(1.05)	(0.74)
	Total	81	3.75	3.65	3.07	2.87	2.31	2.01
			(0.67)	(0.65)	(1.22)	(1.04)	(1.02)	(0.82)
High	No	51	4.1	4.03	3.83	3.46	1.94	1.68
			(0.50)	(0.58)	(1.02)	(0.87)	(0.85)	(0.73)
	Yes	34	3.9	3.91	3.18	3.19	2.14	1.57
			(0.58)	(0.60)	(1.23)	(1.15)	(0.84)	(0.57)
	Total	85	4.02	3.98	3.57	3.35	2.02	1.64
			(0.54)	(0.59)	(1.15)	(0.99)	(0.85)	(0.67)

Possible ratings on all measures range from 1 to 5. Higher mean scores indicate higher positive affect, vitality, and anxiety. The ratings in this table are the uncorrected descriptive statistics and may hence differ from the estimated marginal means reported in the main text.

#### Positive affect

**Fig 2:**
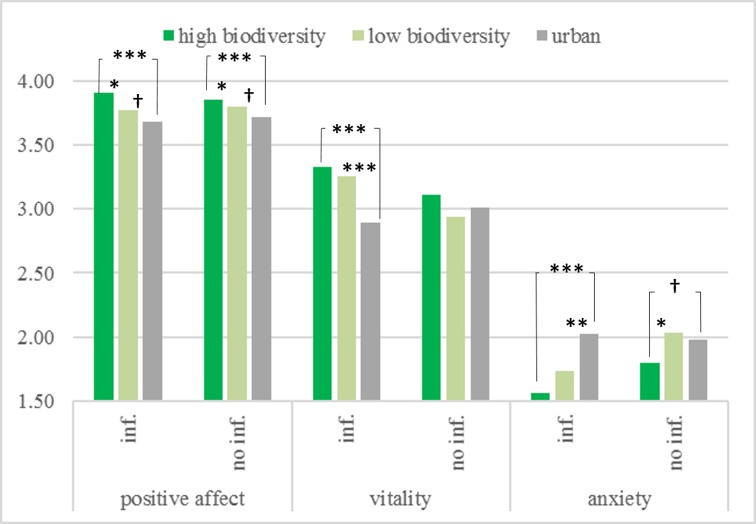
Mean ratings for environment and information factors on positive affect, vitality, anxiety in Study 2. All means are estimated marginal means at follow-up, adjusted for baseline. Possible ratings on all measures range from 1 to 5. Higher mean scores indicate higher positive affect, vitality, and anxiety. † marginally significant at .10 level * significant at .05 level ** significant at .01 level *** significant at .001 level.

Environment significantly shaped positive affect (*F*[2,257] = 7.92, *p <* .001, *η*^*2*^ = .06). As can be seen in Fig 2, contrasts comparing pairs of treatments showed that participants in the high biodiversity treatment indicated more positive affect (*M =* 3.88, *SE =* 0.03) than participants in the low biodiversity treatment (*M =* 3.78, *SE =* 0.03; *p =* .038, CI 95% for difference [-0.19, -0.01]), and in the urban built treatment (*M =* 3.70, *SE =* 0.03; *p <* .001, CI 95% for difference [-0.27, -0.09]). Participants in the low biodiversity treatment reported marginally more positive affect than participants in the urban built treatment (*p =* .077, CI 95% for difference [-0.17, 0.01]). The effect of information, (*F*[1,257] = 0.02, *p =* .89, *η*^*2*^ = .00) and its interaction with environment (*F*[2,257] = 0.78, *p =* .46, *η*^*2*^ = .01) were non-significant.

#### Vitality

The ANCOVA with vitality as the outcome variable revealed a significant effect of environment (*F*[2,257] = 6.48, *p =* .002, *η*^*2*^ = .05). Participants in the high biodiversity treatment reported higher vitality (*M =* 3.22, *SE =* 0.06) than those in the urban treatment (*M =* 2.95, *SE =* 0.05; *p <* .001, CI 95% for difference [-0.42, -0.12]), but did not differ significantly from those in the low biodiversity treatment (*M =* 3.10, *SE =* 0.03; *p =* .14, CI 95% for difference [-0.28, 0.04]). Participants who viewed the low biodiversity video reported higher vitality than those who viewed the urban video (*p =* .048, CI 95% for difference [-0.31, 0.00]). Information also had a significant effect on vitality (*F*[2,257] = 4.86, *p =* .028, *η*^*2*^ = .02) with participants who received information reporting higher vitality (*M =* 3.16, *SE =* 0.04) than participants who did not (*M =* 3.02, *SE =* 0.04; CI 95% for difference [-0.27, -0.02]).

Moreover, environment type interacted with the information treatment (*F*[2,257] = 4.50, *p =* .012, *η*^*2*^ = .03). Breaking this interaction down revealed that environment impacted vitality in the information treatment (*F*[2,126] = 13.74, *p <* .001, *η*^*2*^ = .18) but not in the no information treatment (see Fig 2; *F*[2,130] = 1.50, *p =* .23, *η*^*2*^ = .02). For those who were informed only, viewing a high biodiversity video led to higher vitality (*M =* 3.32, *SE =* 0.08) than viewing an urban video (*M =* 2.85, *SE =* 0.06; *p <* .001, CI 95% for difference [-0.67, -0.27]), while there was no difference between high and low biodiversity (*M =* 3.25, *SE =* 0.07; *p =* .55, CI 95% for difference [-0.28, 0.15]). Moreover, participants viewing the low biodiversity video reported higher vitality than those viewing the urban video (*p <* .001, CI 95% for difference [-0.60, -0.21]).

To look at the simple effects of information, participants in the high biodiversity treatment who received information reported higher vitality (*M =* 3.50, *SE =* 0.08) than those who did not (*M =* 3.25, *SE =* 0.07; *p =* .018, CI 95% for difference [0.05, 0.46]). Similarly, participants in the low biodiversity treatment who received information reported higher vitality (*M =* 3.03, *SE =* 0.09) than those who did not (*M =* 2.72, *SE =* 0.09; *p =* .011, CI 95% for difference [0.07, 0.56]). However, there was no effect of information in the urban built treatment (*p =* .34, CI 95% for difference [-0.33, 0.11]).

#### Anxiety

Environment had a significant effect on reported anxiety (*F*[2,257] = 13.30, *p <* .001, *η*^*2*^ = .09). In particular, participants in the high biodiversity treatment reported lower anxiety (*M =* 1.68, *SE =* 0.05) than those in the low biodiversity treatment (*M =* 1.88, *SE =* 0.05; *p =* .002, CI 95% for difference [0.08, 0.34]), and those in the urban built treatment (*M =* 2.00, *SE =* 0.04); *p <* .001, CI 95% for difference [0.20, 0.44]). Participants in the low biodiversity treatment reported marginally lower anxiety than those in the urban built treatment (*p =* .063, CI 95% for difference [-0.01, 0.24]). Moreover, the effect of information was significant (*F*[1,257] = 10.71, *p =* .001, *η*^*2*^ = .04), indicating that the information treatment lowered anxiety (*M =* 1.77, *SE =* 0.04) as compared to the no information treatment (*M =* 1.94, *SE =* 0.04; CI 95% for difference [0.07, 0.27]).

The information treatment shaped the relation between environment type and anxiety (interaction *F*[2,257] = 4.18, *p =* .016, *η*^*2*^ = .03). The simple effects of environment were significant both in the information treatment (*F*[2,126] = 12.72, *p <* .001, *η*^*2*^ = .17) and in the no information treatment (see Fig 2; *F*[2,130] = 3.52, *p =* .033, *η*^*2*^ = .05). In the information treatment, participants who saw the high biodiversity video reported lower anxiety (*M =* 1.54, *SE =* 0.07) than those who saw the urban video (*M =* 1.98, *SE =* 0.06; *p <* .001, CI 95% for difference [0.27, 0.62]), and marginally lower anxiety than those who saw the low biodiversity video (*M =* 1.72, *SE =* 0.07; *p =* .056, CI 95% for difference [-0.01, 0.38]). Those who viewed the low biodiversity video reported lower anxiety than those who viewed the urban video (*p =* .004, CI 95% for difference [0.43, 0.09]). In the no information treatment, participants who saw the high biodiversity video reported lower anxiety (*M =* 1.83, *SE =* 0.06) than those who saw the low biodiversity video (*M =* 2.05, *SE =* 0.07; *p =* .013, CI 95% for difference [0.05, 0.40]), and marginally lower anxiety than those who saw the urban video (*M =* 2.00, *SE =* 0.07; *p =* .058, CI 95% for difference [-0.01, 0.34]). Participants who saw the low biodiversity video did not differ from those who saw the urban video (*p =* .56, CI 95% for difference [-0.24, 0.13]).

To look at the simple effects of information, participants in the high biodiversity treatment who received information reported lower anxiety (*M =* 1.50, *SE =* 0.08) than those who did not (*M =* 1.73, *SE =* 0.06; *p =* .027, CI 95% for difference [0.03, 0.42]). Similarly, participants in the low biodiversity treatment who received information reported lower anxiety (*M =* 1.86, *SE =* 0.06) than those who did not (*M =* 2.17, *SE =* 0.06; *p <* .001, CI 95% for difference [0.14, 0.47]). However, there was no effect of information in the urban built treatment (*p =* .45, CI 95% for difference [-0.24, 0.11]).

#### Change in well-being from baseline

Finally, we looked at the effects of environment in more detail by examining participants’ change in well-being from the baseline measures to the follow-up measures. We conducted a 3 (environment: urban built vs. low biodiversity vs. high biodiversity) x 2 (measurement moment: baseline vs. follow-up) MANCOVA on positive affect, vitality, and anxiety. The crucial interaction between environment and measurement moment was significant for positive affect (*F*[2,261] = 6.74, *p* = .001, *η*^*2*^ = .05), vitality (*F*[2,261] = 5.09, *p* = .007, *η*^*2*^ = .04), and anxiety (*F*[2,261] = 7.88, *p* < .001, *η*^*2*^ = .06).

**Fig 3:**
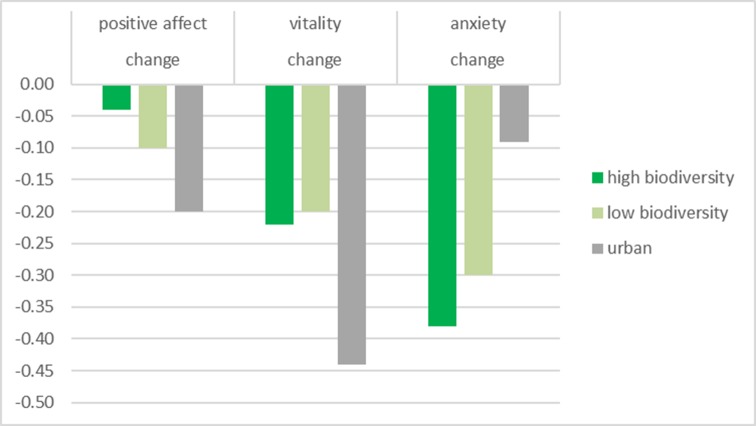
Mean change from baseline to follow-up for environment factor on mental well-being in Study 2. Possible ratings on all measures range from -5 to +5. Higher negative scores indicate greater reductions from baseline to follow-up in positive affect, higher vitality, anxiety. With the exception of positive affect in the high biodiversity treatment, all means are significantly below zero.

Examining this interaction effect in the urban treatment revealed that participants’ positive affect (*F*[1,97] = 39.74, *p* < .001, *η*^*2*^ = .29) and vitality (*F*[1,97] = 55.44, *p* < .001, *η*^*2*^ = .36) decreased strongly from baseline to follow-up. Anxiety decreased only weakly (*F*[1,97] = 3.95, *p* = .050, *η*^*2*^ = .04). In the low biodiversity treatment, participants’ positive affect (*F*[1,80] = 8.25, *p* = .005, *η*^*2*^ = .09) and vitality (*F*[1,80] = 6.97, *p* = .010, *η*^*2*^ = .08) also decreased, but to a weaker extent. In contrast, participants’ anxiety was strongly reduced at follow-up (*F*[1,80] = 27.39, *p* < .001, *η*^*2*^ = .26). For participants in the high biodiversity treatment, positive affect did not decrease significantly (*F*[1,84] = 1.44, *p* = .23, *η*^*2*^ = .02) while anxiety decreased strongly (*F*[1,84] = 38.26, *p* < .001, *η*^*2*^ = .31). However, participants’ vitality also decreased in the high biodiversity treatment (see Fig 3; *F*[1,84] = 13.93, *p* < .001, *η*^*2*^ = .14).

## Study 2 –Summary

Study 2 manipulated biodiversity by comparing the effect of viewing built environments with viewing natural environments high or low in bird species diversity. We found that participants who were exposed to natural environments low in biodiversity reported higher vitality and tended to report more positive affect and lower anxiety than participants who were exposed to urban built environments. Hence, even natural environments low in biodiversity are beneficial for people’s mental well-being compared to urban environments. Importantly, the data indicated that biodiversity provides additional benefits for people’s mental well-being. That is, participants who were exposed to videos of natural environments high in biodiversity reported higher positive affect and lower anxiety than participants who were exposed to videos of natural environments low in biodiversity. Additionally, they reported higher vitality, a sense of energy and aliveness, than participants who were exposed to urban built environments.

Interestingly however, the average ratings generally decreased between pre- and post-measures in all three treatments, potentially as a result of taking part in the survey. Nevertheless, while participants exposed to urban built environments showed a large drop in their reported positive affect and vitality, participants exposed to natural environments low in biodiversity showed much weaker reductions on these mental well-being indicators. Importantly, only participants exposed to natural environments high in biodiversity showed signs of retaining a high well-being such that they did not report lower positive affect at follow-up. Hence, being exposed to this environment may have buffered their mental well-being against negative affect. Similarly, whereas participants in the urban built treatment showed only a small reduction in anxiety, participants exposed to the natural environments reported much lower anxiety levels at follow-up compared to baseline.

Overall, these findings replicate and extend the results from Study 1 by showing that natural environments high in biodiversity improve people’s mental well-being relative to natural environments low in biodiversity and also relative to built environments. However, it is noteworthy that the effects of environment on vitality and anxiety were dependent on whether participants received information about the environments. That is, the benefits on vitality were only apparent when individuals received information about the environments. Moreover, the beneficial effect of high biodiversity versus low biodiversity on anxiety only emerged when participants did not receive information about the environments. When participants received information, they reported lower anxiety in both natural environments than in the built environment. Conversely, giving participants information about their environments resulted in higher vitality and lower anxiety, but only for participants who were presented with either of the two natural environments–not for those presented with urban built environments. Thus, providing people with information about natural environments seems to improve their mental well-being.

## Discussion

Across two studies, we identified that exposure to higher levels of biodiversity, in terms of species richness while controlling for species abundance, causes an improvement in people’s mental well-being. In particular, people exposed to natural environments higher in biodiversity consistently reported higher positive affect and lower anxiety afterwards than people who viewed natural environments low in biodiversity or people who viewed urban built environments. We obtained effects of biodiversity on mental well-being whether using trees or birds, suggesting our results may be generalizable across different flora and fauna.

The present study extends past research linking biodiversity to people’s mental well-being in several ways. First, to the best of our knowledge, previous work, both correlational and experimental, has confounded species richness and species abundance, and thus, the present study is among the first to demonstrate a causal role of species richness, controlling for species abundance and other confounding factors, in increasing people’s mental well-being. Second, in the impactful study by Fuller et al. [10], mental well-being was assessed in indirect ways and in connection with a particular greenspace. In contrast, the present research examined mental well-being more directly and generally by assessing the widely-used well-being indicators positive affect, vitality, and anxiety, and showing that biodiversity improves a range of mental well-being outcomes.

In Study 2, we also explored the role of giving people information about natural and urban built environments. When information was given, people reported higher vitality when they were exposed to a high biodiversity environment compared to an urban built environment. In contrast, this effect was non-significant when no information was given. Moreover, people who were presented with environments high or low in biodiversity reported higher vitality and lower anxiety when they received information about these environments than when they did not. This effect was not present for information about urban built objects. Hence, this explorative data indicates that giving information may improve mental well-being in response to natural environments.

### Limitations and future research

One potential limitation of this study is that it was conducted via the online platform Mechanical Turk. While there is evidence supporting the high data quality provided by Mechanical Turk respondents [41–43], it may be the case that effects would have been stronger for participants supervised in a lab experiment where all other environmental conditions could be held constant. Another potential limitation is that the selected bird species in Study 2 are all native in Europe and hence the recruited US participants may have been less familiar with them. It is conceivable that more familiar bird species would have improved participants’ mental well-being more, similar to the beneficial effect of giving participants information on the bird species. As well, other demographics such as socioeconomic status or place of residence (e.g., urban vs. rural dwellers) might impact the present findings, and future research using sufficiently robust sample sizes should examine these as potential moderators of the present findings.

Future research could also extend the present findings by investigating whether people benefit more from urban greenspaces high in biodiversity than from urban greenspaces low in biodiversity. For example, research might test whether there are well-being effects of exposure to such elements as planters, gardens, butterflies, or birds at feeders in these urban spaces. These studies would be especially informative for urban planners who may not be able to integrate ‘wild’ nature into city spaces, and may also inform how the well-being of urban residents is at present enhanced by natural elements around them. In addition, building on the present findings that biodiversity can improve mental well-being, future research could test whether biodiversity alleviates perceptions and physiological indicators of stress (e.g., heart rate, skin conductance, blood pressure) [37] in participants. For example, biodiversity could be manipulated following a stress manipulation in a 2 X 2 design to examine changes from baseline to follow-up.

Finally, given that our high biodiversity treatment contained only four to five species, it would be useful for future studies to test the effects of environments that are higher in biodiversity on mental well-being. Indeed, many ecosystem functions and ecosystem services increase with species richness but plateau at around 10–20 species [56–58]. Moreover, past research indicates that densely vegetated (and hence perhaps high biodiversity) environments may be detrimental to mental well-being because they could be seen as more threatening [59–61]. Thus, future research could benefit from examining people’s mental well-being at higher levels of biodiversity than those examined here.

### Conclusion

Overall, the present research presented experimental evidence that both plant and animal biodiversity has a beneficial effect on people’s mental well-being. These studies inform arguments for maintaining plant and animal species—not only for moral reasons or because biodiversity provides us with sufficient resources to sustain physical well-being, but also because our mental well-being benefits from natural environments that are rich in animal and plant species. Given ongoing urbanisation [1] as well as biodiversity loss [3–5], our results argue for the importance not just of maintaining biodiversity at a global scale, but of retaining and enhancing biodiverse environments in or near to towns and cities, and of ensuring people have both the physical access and the information they need to benefit from it.

## Supporting Information

S1 Data Dataset Study 1. (SAV)

S2 Data Dataset Study 2. (SAV)
